# Health Tract, No. 234

**Published:** 1865

**Authors:** 


### PUNCTUALITY.

"The want of punctuality is the want of virtue," was a favorite saying of our sterling old Puritan minister, the Rev. John McFarland, the model pastor of his time, when he wished to drum us all up, not only to attend the "Catechism class" every Saturday afternoon, but to have us present at the very minute, so as not keep the more punctual ones waiting. "You have no right," he used to say, "by being a minute too late yourself, to make another lose that minute by waiting for you; and that minute multiplied by all the members, makes a frightful loss of time; time which you have caused them to lose, without any benefit to yourself." What a trainer of children and youth and men he was! Nearly every boy in that "Catechism class" became a clergyman or a missionary; and every girl, not a name missing, either married a clergyman or lived to be "a mother in Israel;" there is Polly Hall, and Prudence Lilly, and Ann Alexander, and Jane Hamilton, and Isabella Sharpe, and others, with their bright, black eyes and sweet voices and dear, loving, friendly hearts. It is more than forty years agone now, and most of them are in heaven to-day; here and there one is left, but they are all blossoming for immortality; soon to be transplanted into the garden of the blessed, and by the still waters of the upper Eden, to grow and thrive forever!

But the boys, where are they? Of a large class, all but three, as we remember to-day, became ministers or stood high among "the men of their time." Some are standing still, flourishing like green bay-trees; the shadows of their influence spreading across seas and continents. There's Harvey Lilly, and Thornton Mills, and William Alexander, and James Hamilton, and William Lyle, and Thomas Sharpe, and Robert Bishop, and a long list of other names of those who became good men and true, under Mr. McFarland's teaching in the Catechism and the Bible-class. The three exceptions never could be induced to attend with any kind of regularity; they took but little interest in psalms and hymns and spiritual songs; they all fill now dishonored graves; one died a drunkard; one shot his brains out; the third became the fear of the town, killed two men, was condemned to be hung, and to avoid it, killed himself.

Such are the results, in a moral point of view, of punctuality, or the want of it, in attention to the duties of life; and like high benefits will result physiologically to those who are punctual, regular, systematic, in all their bodily habits, of eating, sleeping, bathing, exercise, study, and recreation. Those who begin these things early in life, have the like characteristics to pervade their mental and moral nature, and grow up to be healthful in body, vigorous in mind, and reliable in society; they become men who can be depended on; you always know where to find them; in all their opinions they are clear, sharp, definite; are the pride of their parents, and an honor to any community.

